# Aging Adipose‐Derived Mesenchymal Stem Cells, Cultured on a Native Young Extracellular Matrix, Are Protected From Senescence and Apoptosis Along With Increased Expression of HLA‐DR and CD74 Associated With PI3K Signaling

**DOI:** 10.1111/acel.70165

**Published:** 2025-08-05

**Authors:** Aaron O. Gonzalez, Parveez A. Abdul Azees, Jerry P. Chen, Milos Marinkovic, Brian Cao, Ting Liang, Peiqing Hu, Chih‐Ko Yeh, David D. Dean, Yidong Bai, Xiao‐Dong Chen

**Affiliations:** ^1^ Department of Comprehensive Dentistry, UT Health San Antonio School of Dentistry San Antonio Texas USA; ^2^ Department of Biomedical Engineering University of Texas at San Antonio San Antonio Texas USA; ^3^ School of Dentistry, The Center for Regenerative Sciences UT Health San Antonio San Antonio Texas USA; ^4^ Research Service, South Texas Veterans Health Care System Audie Murphy VA Medical Center San Antonio Texas USA; ^5^ Department of Cell Systems and Anatomy, UT Health San Antonio Long School of Medicine San Antonio Texas USA; ^6^ Geriatric Research, Education and Clinical Center, South Texas Veterans Health Care System Audie Murphy VA Medical Center San Antonio Texas USA

**Keywords:** aged MSCs, autologous stem cell therapies, CD74, immunogenicity, MHC class II, young microenvironment

## Abstract

Older adults are the primary population for cell‐based therapies for age‐related diseases, but the efficacy of administering autologous mesenchymal stem cells (MSCs) is impaired due to biological aging. In the present study, we cultured aging adipose (AD)‐derived MSCs from > 65‐year‐old donors on extracellular matrix (ECM) synthesized by human amniotic fluid‐derived pluripotent stem cells (ECM Plus) versus tissue culture plastic (TCP) and hypothesized that ECM Plus provided an ideal “young” microenvironment for reactivating and preserving early‐stage progenitor cells within aging AD‐MSCs. To test our hypothesis, we serially sub‐cultured aging AD‐MSCs on ECM Plus or TCP and characterized the cells both phenotypically and functionally, and then analyzed the cells at the single‐cell transcriptomic level for the mechanisms that control cell fate. The results showed that the maintenance of aging AD‐MSCs on ECM Plus significantly restored their quantity and quality. The mechanisms responsible for these effects were associated with a remarkable up‐regulation of intracellular CD74 when cells were maintained on ECM Plus compared to TCP, which triggered activation of the phosphoinositide‐3‐kinase (PI3K) pathway as a key modulator of cell survival (anti‐apoptosis) and suppression of cellular senescence. Moreover, AD‐MSCs maintained on ECM Plus increased their expression of HLA‐DR and stimulated T cell activity. These findings challenge the “immune privilege” of allogeneic MSCs as a universal source for MSC‐based therapies. The present study leads to a new paradigm for treating age‐related diseases: serial administration of rejuvenated autologous MSCs, which may not only replace aged MSCs but also gradually reverse the aged microenvironment.

## Graphical abstract

The maintenance of aging mesenchymal stem cells (MSCs) on extracellular matrix synthesized by human amniotic fluid‐derived pluripotent stem cells (ECM Plus), a young microenvironment, remarkably restored their quantity and quality along with up‐regulating HLA‐DR and CD74. The former stimulated T cell activity and the latter triggered PI3K signaling protecting cells from cellular senescence and apoptosis. The present study challenges the “immune privilege” of allogeneic MSCs and provides a new paradigm of autologous MSC‐based therapies for age‐related diseases.

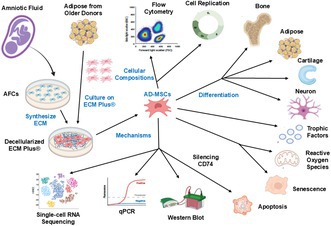

## 1 Introduction

Mesenchymal stem cells (MSCs) hold immense promise as regenerative therapies due to their multipotent differentiation potential and ability to produce anti‐inflammatory and immunomodulatory trophic/growth factors (Caplan 2017). Since MSCs expanded in vitro do not express human leukocyte antigen (HLA)‐DR, proponents of the “universal donor” paradigm maintain that allogenic MSCs, as a “one‐size‐fits all” source, be used for MSC‐based therapies. However, MSC immune privilege has been challenged by mounting evidence suggesting that allogenic MSCs are capable of stimulating an immune response that ultimately leads to rejection (Ankrum et al. 2014). While MSCs produce trophic factors that potently modulate the immune system, they can also be affected by the local inflammatory environment or induced to differentiate into mature cells expressing high levels of MHC class I and II antigens (Le Blanc et al. 2003), resulting in delayed rejection of the allogenic MSCs by the host immune system. Moreover, transplanted allogenic MSCs are more rapidly rejected in previously sensitized animals, with most cells killed within 48 h after systemic infusion (Zangi et al. 2009). This observation is consistent with the fact that very few studies show transplanted allogeneic MSCs directly forming new tissue in vivo (Caplan 2017), as opposed to these cells secreting trophic factors that reactivate the quiescent endogenous stem cell population (Munoz et al. 2005). When biosafety concerns related to the transmission of pathogens (known and unknown) from the transplanted allogenic MSCs to the recipient are taken into consideration, autologous stem cell‐based therapies are preferable.

Older adults are the primary population for cell‐based treatment of age‐related diseases, but the efficacy of their MSCs is diminished due to the adverse effects of biological aging (Block et al. 2017). To improve the quantity and quality of MSCs, we previously described the preparation and characterization of a novel cell culture system which is based on a native three‐dimensional (3D) decellularized bone marrow‐derived extracellular matrix (BM‐ECM) (Chen et al. 2007). This system provides many of the critical biochemical, architectural, and physical/mechanical cues needed for retaining the stemness of MSCs in vitro relative to traditional tissue culture plastic (TCP) (Chen et al. 2007; Lai et al. 2010; Marinkovic et al. 2016). Recently, Block et al. developed ECM Plus (StemBioSys Inc., San Antonio, TX), which is synthesized by human amniotic fluid‐derived pluripotent stem cells (AFCs), to maintain human induced pluripotent stem cell‐derived cardiomyocytes (hiPSC‐CMs) in vitro (Block et al. 2020). ECM Plus primarily consists of collagens, glycoproteins, and basement membrane proteins, which represent critical components of the stem cell niche (Block et al. 2020). Indeed, we found that ECM Plus also promoted the proliferation and differentiation capacity of BM‐MSCs and adipose‐derived MSCs (AD‐MSCs) (unpublished data from the Chen lab).

Autologous AD‐MSCs are a practical source of therapeutic cells for aging patients because they display many of the same features as BM‐MSCs (reviewed in (Feisst et al. 2015)) and offer the following unique advantages: (i) adipose tissue is a richer source of stem cells than BM (~0.39% vs. ~0.002%, respectively) (Kern et al. 2006), (ii) harvesting adipose tissue is less invasive than BM, and (iii) adipose tissue is one of the largest tissues in the body based on mass (> 5% [men] or 10% [women] for an adult human body) and therefore provides an abundant, practical, and attractive source of tissue for obtaining autologous MSCs. However, AD‐MSCs have been shown to favor adipogenesis over osteogenesis in vivo (Hayashi et al. 2008). Thus, to pursue autologous AD‐MSC‐based therapies in aging patients, we must first rescue the quantity and quality of the stem cells and then guide their differentiation to the desired cell lineage, such as osteoblasts.

In the present study, we cultured aging AD‐MSCs, obtained from > 65‐year‐old donors, on ECM Plus and hypothesized that ECM Plus, synthesized by AFCs, provided an ideal “young” microenvironment for preserving and amplifying early‐stage progenitor cells within the aging AD‐MSCs. To test our hypothesis, we serially sub‐cultured aging AD‐MSCs on ECM Plus versus TCP and then characterized the cells both phenotypically and functionally; subsequently, we analyzed the cells at the single‐cell transcriptomic level to understand the underlying molecular mechanisms for controlling cell fate. The results showed that maintenance of aging AD‐MSCs on a “young” microenvironment (i.e., ECM Plus) restored their capacity for self‐renewal, differentiation, and in vivo bone formation (vs. adipose tissue). More importantly, ECM Plus promoted an antigen‐presenting phenotype, involving the increased expression of HLA‐DR, which activated T cells, and CD74, which inhibited cellular apoptosis via the phosphoinositide 3‐kinase (PI3K) pathway.

## 2 Materials and Methods

### 2.1 Culture of MSCs

Aging AD‐MSCs (> 65 year‐old male and female donors, passage (P) 2 or 3) and umbilical cord or Wharton's Jelly (WJ) (< P5) cells were obtained from StemBioSys Inc. (San Antonio, TX). Cells were cultured at 37°C, 5% CO_2_, and 100% humidity in growth media (phenol red‐free α‐MEM containing 2 mM L‐glutamine and 10% fetal calf serum [FBS]). For each experiment, MSCs were seeded at 4000 cells/cm^2^ onto TCP or ECM Plus (StemBioSys, San Antonio, TX), which was prepared as previously described (Block et al. 2020), and cultured for 7 days (~80% confluence) for the indicated assays or serially sub‐cultured. For each passage, the doubling time (*DT*) and cumulative population doublings (*PD*) for each substrate were computed as previously described (Bonab et al. 2006).

Theoretically, we should have used youthful AD‐MSCs as controls for aging AD‐MSCs. However, young donors have been shown to display large variations in AD‐MSC quality due to differences in gender, body mass index, donor collection site (e.g., abdomen, waist), and preparation method (Tsekouras et al. 2017; Gentile et al. 2019). In contrast, WJ cells consistently display a youthful phenotype and were used as a surrogate for “young” MSCs in the present study.

In addition, we selected TCP as a “negative control”, because it is impossible to design an “old”‐ECM made by “old” cells from the same type of tissue (i.e., human amniotic fluid‐derived pluripotent stem cells) used to produce ECM Plus. On the other hand, it would be inappropriate to compare “old” BM‐ECM with ECM Plus since different tissue types of ECMs have tissue‐specific effects on MSC fate (Marinkovic et al. 2016).

### 2.2 Surface Marker Analysis Using Flow Cytometry

Cells were suspended in staining buffer (PBS containing 5% FBS and 0.1% (w/v) sodium azide) and individually incubated overnight at 4°C with the following primary antibodies obtained from BD‐Bioscience against: SSEA4, HLA‐DR, CD90, CD74, CD146, CD80, CD86, or isotype‐matched controls. The cells were then rinsed and incubated for 1 h with FITC‐conjugated goat anti‐mouse IgG/IgM (BD‐Bioscience) in the dark at 4°C. Next, the cells were rinsed, fixed with 2% paraformaldehyde, and analyzed within 24 h using the AF488 channel in the BD FACS Celesta Cell Analyzer. Cells were gated based on doublet exclusion.

### 2.3 Telomere Length Measurement

Telomere length was analyzed by real‐time qPCR. Genomic DNA was extracted from MSCs subcultured on ECM Plus or TCP using a DNeasy Blood &Tissue Kit (QIAGEN) and then 20 ng was amplified using SYBR Green PCR Master Mix (Qiagen) and a 7900HT Fast Real‐Time PCR System (Applied Biosystems). Telomere length was calculated as previously described (Lin et al. 2019).

### 2.4 ROS Quantification

MSCs (3 × 10^5^) expanded on TCP or ECM Plus were transferred to 1.5 mL tubes and incubated at 37°C to quantitate the presence of ROS using the DCFDA Cellular ROS Assay Kit (Abcam, Boston, MA). After 4 h, cells were immediately analyzed with the BD FACS Celesta Cell Analyzer using the AF488 channel and gated based on doublet exclusion.

### 2.5 β‐Galactosidase (β‐Gal) Quantification

MSCs expanded on TCP or ECM Plus were seeded at high density (25,000 cells/cm^2^) in 6‐well plates overnight and then stained for β‐gal using the Spider β‐gal Senescence Detection Kit (Dojindo, Rockville, MD) for 1 h at 37°C. Following treatment, the cells were detached, switched to flow cytometry staining buffer, and analyzed with the BD FACS Celesta Cell Analyzer using the AF488 channel and gated based on doublet exclusion.

### 2.6 Apoptosis Detected by TUNEL Assay

Aging AD‐MSCs were cultured on TCP or ECM Plus for 7 days and apoptotic cells were identified by TUNEL (TdT‐mediated dUTP nick end labeling) assay using the In Situ Cell Death Detection Kit (Roche, Germany), according to the manufacturer's instructions. Apoptotic cells were visualized by fluorescence microscopy. Images were acquired and analyzed using NIH Image J. For each sample, 5 random fields were selected. Fluorescence intensity was quantified by calculating the mean gray value, which represents the sum of intensity values within the region of interest (ROI) divided by the total number of pixels in that area. Results were expressed as a percentage relative to the total fluorescent area (Schneider et al. 2012).

### 2.7 Colony‐Forming Unit Assays

MSCs serially sub‐cultured on either TCP or ECM Plus were detached and seeded into TCP 6‐well plates at 600 and 200 cells/well for colony‐forming unit (CFU)‐ osteoblast (OB) and ‐adipocyte (AD) assays and 300 and 100 cells/well for CFU‐fibroblast (F) assay. Following a 7‐day incubation period, cell culture media were replenished every 72 h. After 14 days in culture, CFU‐F colonies were visualized by crystal violet staining. To assess CFU‐OB colony formation, CFU‐F colonies were maintained for an additional 25 days in osteoblast differentiation media (growth media supplemented with 10^−7^ M dexamethasone [Sigma] and 10^−4^ M L‐ascorbate‐2‐phosphate [Wako Chemicals, Richmond, VA]) and CFU‐OB colonies were detected by von Kossa staining and quantified using NIH Image J. To assess CFU‐AD colony formation, CFU‐F colonies were maintained for an additional 10 days in adipogenic media (DMEM containing 10% FBS, 0.5 mM IBMX, 10^−6^ M dexamethasone, 10 μM insulin, 200 μM indomethacin) and CFU‐AD colonies were visualized by Oil Red O staining. A complete description of the CFU assays has been previously published (Lai et al. 2010).

### 2.8 Chondrogenic Differentiation

MSCs cultured on TCP or ECM Plus were re‐suspended at 10^6^ cells/mL in either control media (DMEM containing 2 mM L‐glutamine and 10% FBS) or Promocell Chondrogenic Differentiation Medium (Fisher Scientific, Waltham, MA) and seeded into U‐Bottom Suspension 96‐well plates (Genesee Scientific, San Diego, CA). During 21 days of culture, half media changes were performed every 72 h. At harvest, cell pellets were collected and prepared for imaging, histological analysis, or qPCR.

For image analysis, cell pellets were fixed for 2 h in 10% formalin and re‐suspended in 1X PBS. Brightfield images of the cell pellets were captured using NIS‐Elements software on a Nikon Eclipse Ti inverted microscope with a 10X/NA 0.45 objective and Ri2 Camera in the UT Health San Antonio Optical Imaging Core, and the images were color‐inverted using Image J.

For histological analysis, formalin‐fixed cell pellets were transferred to 1.5 mL centrifuge tubes, 3% aqueous agar was slowly added, and then allowed to harden. The samples were bisected, placed into cassettes, and then transferred to 70% ethanol for storage. Samples were further processed and stained with H&E and Alcian Blue in the Department of Pathology and Lab Medicine (UT Health San Antonio).

### 2.9 Neurogenic Differentiation

MSCs cultured on TCP or ECM Plus were detached and seeded at high density (25,000 cells/cm^2^) in TCP 6‐well plates. After 24 h, cells were switched to either control MSC Growth Medium 2 (Promocell) or Neurogenic Differentiation Medium (Promocell) and maintained for 3–10 days, depending on the subsequent assay. Images of the MSCs were taken at 24 h post‐induction to observe morphological changes, while RNA isolation and Nissl body staining were performed at Days 9–10 as previously described (De Simone et al. 2019). Briefly, cells were fixed for 2 h with 10% formalin, rinsed twice with PBS, and 0.5% aqueous cresyl violet (Sigma Aldrich) added to each well. Following incubation for 30 min at room temperature, the samples were rinsed multiple times with PBS and visualized under a brightfield microscope. Nissl bodies were identified as regions with a dark purple hue.

### 2.10 Assessment of Anti‐Inflammatory Response

MSCs expanded on TCP or ECM Plus were seeded at 25,000 cells/cm^2^ into TCP 6‐well plates in growth media, incubated overnight, and then switched to serum‐free growth media. After a 24‐h incubation period, cells were switched to serum‐free growth media containing 20 ng/mL of human recombinant TNF‐α (Peprotech, Rocky Hill, NJ) or vehicle and incubated for an additional 48 h prior to gene expression analysis.

### 2.11 Gene Expression Analysis Using Real Time PCR

Cells were rinsed once with cold PBS and stored in Trizol (Invitrogen, Waltham, MA) at −80°C. RNA was purified using the chloroform extraction method, as recommended by the manufacturer. The purified RNA was used to produce 1 μg cDNA using a High‐Capacity cDNA Archive Kit (Applied Biosystems, Foster City, CA). Transcripts of interest, as well as that of the housekeeping gene (GAPDH), were amplified from the cDNA by real‐time PCR using TaqMan Universal PCR Master Mix and Assay‐on‐Demand or Assay‐by‐Design primer/probe sets (Applied Biosystems). Amplification and detection were carried out with an ABI 7900HT Fast Real‐Time PCR System (Applied Biosystems). Gene expression was quantified by subtracting the GAPDH threshold cycle (Ct) value from the Ct value of the gene of interest and expressed as 2^−ΔCt^ as described by the manufacturer's protocol.

### 2.12 In Vivo Bone Formation

Cells (1 × 10^6^) pre‐cultured on TCP or ECM Plus were loaded into a transplantation vehicle [hydroxyapatite/tricalcium phosphate (HA/TCP) powder, Zimmer Inc., Warsaw, IN, USA] and implanted subcutaneously into the dorsal surface of 10‐week‐old immunodeficient beige mice (NIH‐bg‐nu‐xid, Harlan Sprague Dawley, Indianapolis, IN) as previously described (Bi et al. 2005). Three implants were prepared for each condition, harvested after 8 weeks, fixed in 10% phosphate buffered formalin at 4°C for 24 h., decalcified in 10% EDTA (pH 8.0) at room temperature for 1–2 weeks, and then embedded in paraffin. Each ossicle was bisected by collecting 10 μm thick sections at 100 μm intervals. A total of 9 hematoxylin–eosin (H&E) stained sections were used for quantification. Bone‐like regions (i.e., highly stained ECM containing osteocytes; not HA) in each section were measured by NIH Image J software and summed. Then, the total area of bone‐like regions was divided by the entire area of the section (which included bone‐like tissue, fibrous connective tissue, HA, adipose tissue, etc.).

### 2.13 Bioenergetics Measurements

As previously described (Liang et al. 2022; Qin et al. 2024), aging AD‐MSCs and WJ cells pre‐cultured on either TCP or ECM Plus were plated at a density of 40,000 cells per well in an XF96 cell culture microplate (Seahorse Bioscience) in 200 μL of media and incubated overnight (14–16 h.) at 37°C in a humidified atmosphere of 5% (v/v) CO_2_. One hour prior to performing the assay, the culture media were changed to Seahorse assay media supplemented with 10 mM glucose, 4 mM glutamate, and 1 mM sodium pyruvate, and the plate returned to a 37°C incubator without CO_2_. OCR and ECAR values were measured at baseline and after sequential administration of oligomycin (2.0 μM), FCCP (1.0 μM), and rotenone (0.6 μM)/antimycin A (0.5 μM). Indices of mitochondrial function, including basal respiration, maximal respiration, and coupling efficiency, were then calculated accordingly (Qin et al. 2024).

### 2.14 Blue Native Polyacrylamide Gel Electrophoresis (BN‐PAGE)

BN‐PAGE (3%–11% gradient) was used to separate the respiratory complexes. A ratio of 6 g digitonin/g protein was used to solubilize OXPHOS protein from isolated mitochondria, and 30 μg of mitochondrial protein lysate was loaded onto a 1.5 × 70 × 82 mm mini gel (Bio‐Rad). The gels were run at 5 mA per gel for about 60 min or stopped when the dye reached approximately 1/3 of the separating gel. The cathode buffer was then exchanged for colorless cathode buffer, and the gel was run until the dye reached the end of the gel. Protein complexes were detected by Western blot using the antibodies described previously (Liang et al. 2022).

### 2.15 Single‐Cell RNA Sequencing (RNA‐Seq) Analysis

#### 2.15.1 Sample Preparation for Library Construction

Aging AD‐MSCs pre‐cultured on TCP or ECM Plus were suspended in isolation buffer (PBS containing 5% FBS) at 700–1200 cells/μL and then submitted to the Genomic Sequencing Facility (UT Health San Antonio). The facility followed the 10× Genomics instructions using a single‐cell gene expression V3.1 kit for cell QC and library prep/sequencing with a target count of 5000 cells. Alignment of single‐cell RNA sequencing was performed by the Genomic Sequencing Facility using Cell Ranger and the hg38 reference genome.

#### 2.15.2 Identification and Enrichment of MSC Sub‐Populations

Single‐cell RNA sequencing analysis was conducted using *Seurat*, version 3.1.4, and followed a modified approach for integrated analysis (Stuart and Satija 2019), which was performed by determining the number of features, identifying the integration anchors, and running data integration. The integrated matrix was then scaled and dimensionally reduced using the Uniform Manifold Approximation and Projection for Dimension Reduction (UMAP) technique.

Differentially expressed genes (DEGs) were identified at the sub‐population level as well as at the substrate level. The resulting DEGs were filtered based on an adjusted *p*‐value < 0.05 and logFC ≥ 0.2. The up‐ or down‐regulated genes in each cluster were submitted to the EnrichR web‐based tool (Chen et al. 2013) for gene enrichment analysis. DEGs at the substrate level were submitted to the Search Tool for the Retrieval of Interacting Genes/Proteins (STRING) database to generate candidate signaling networks (Franceschini et al. 2013).

Genes associated with senescence and apoptosis were acquired from the CellAge database (Tacutu et al. 2018) and KEGG_APOPTOSIS gene set (Kanehisa and Goto 2000), respectively. The genes from these lists were compared against the total number of substrate‐level DEGs to identify potential matches. A heatmap against DEGs found in these lists was generated using average subpopulation expression levels.

### 2.16 Isolation and Expansion of HLA‐DR ^+^ MSCs

Aging AD‐MSCs cultured on ECM Plus were serially sub‐cultured for 3–4 passages. After culture, these cells were stained for expression of HLA‐DR and sorted under sterile conditions by the Flow Cytometry Facility (UT Health San Antonio) using a FACSAria III (BD Biosciences). Fractionated HLA‐DR^+^ and HLA‐DR^−^ cells were then serially sub‐cultured on TCP and ECM Plus to monitor changes in their surface and genetic expression of HLA‐DR.

### 2.17 Mixed Lymphocyte Reaction (MLR) Assay

AD‐MSCs pre‐cultured on TCP or ECM Plus were seeded into 6‐well TCP plates at 0.5 × 10^6^ cells/well and then 5 × 10^6^ PBMCs (human peripheral blood mononuclear cells) pre‐labeled with CFSE were added to each well for co‐culture. After 5 days, the cells were incubated with AF647 anti‐human CD4 (Cat# 51‐0049‐42, ThermoFisher) or AF647 isotype‐matched control (Cat# 557732) at room temperature for 30 min. Following multiple rinses, the cells were fixed with 2% paraformaldehyde and processed using the BD FACS Celesta Cell Analyzer, with AF‐488 and AF‐647 channels for determining CFSE and CD4 positive cells, respectively. The dividing T cells were identified by CD4 positive cells with a decreased AF488 (CFSE) intensity.

### 2.18 Western Blot Analysis

Aging AD‐MSCs serially sub‐cultured on either TCP or ECM Plus were harvested and lysed using RIPA buffer containing a commercial protease inhibitor cocktail (Roche, Switzerland). The lysed cells were then centrifuged at 14,000×*g* for 10 min at 4°C, and the supernatant was collected. Protein concentrations were measured using the Pierce BCA Protein Assay Kit (ThermoFisher) and 20–50 μg of extract were resolved using a 4%–20% Mini‐PROTEAN TGX Precast Protein Gel (Bio‐Rad Laboratories, Hercules, CA). The protein was then transferred to a polyvinylidene difluoride (PVDF) membrane. Next, the membranes were incubated in 4% milk blocking solution for 1 h and then probed overnight at 4°C with the following primary antibodies obtained from Cell Signaling Technology Inc. (Danvers, MA): CD74, Runx2, Sox9, Nestin, PI3K, p‐AKT, AKT, and GAPDH. For band visualization, the membranes were incubated with horseradish peroxidase (HRP)‐conjugated secondary antibody for 1 h at room temperature, rinsed three times with TBST, incubated with the chemiluminescent substrate for up to 5 min, and then exposed to X‐ray film. Staining of the bands was quantified using FIJI image analysis software.

### 2.19 Silencing CD74 Expression in Aged AD‐MSCs Maintained on ECM Plus

To silence CD74, a SMARTpool CD74 siRNA (Horizon Discovery, Lafayette, CO) was used. Cells were seeded at 10,000 cells/cm^2^ into ECM Plus 6‐well plates. On Day 3 of culture, ECM Plus‐cultured cells were transfected with the CD74‐targeting siRNA using Lipofectamine and PLUS reagents (Life Technologies), following the manufacturer's protocol. Briefly, for each well of a 6‐well plate, the transfection complex was prepared by combining 5 μL of siRNA (final concentration: 100 nM) and 5 μL of Lipofectamine reagent in 250 μL of Opti‐MEM media (Gibco). The siRNA‐lipid mixture was then added to 750 μL of phenol red‐free α‐MEM containing 15% FBS and incubated with the cells for 48 h. Following transfection, the media were replaced with complete growth medium containing antibiotics. CD74 knockdown efficiency was confirmed by Wb analysis at 72 h post‐transfection.

### 2.20 Statistical Analysis

All data, except for the Seahorse analysis, are presented as the mean ± standard deviation with an *n* of 3–6 depending on the experiment. The Seahorse analysis is presented as a violin plot distribution for *n* = 12 replicates, which was produced using GraphPad Prism v10. Statistical differences were identified using Student's *t*‐test or one‐way ANOVA, with significance set at *p* < 0.05. All experiments were performed using triplicates and repeated in at least 3–6 independent experiments using cells from different donors, including the WJ cells.

## 3 Results

### 3.1 Aging AD‐MSCs Maintained on ECM Plus Display Enhanced Capacity for Proliferation and Differentiation

**FIGURE 1:**
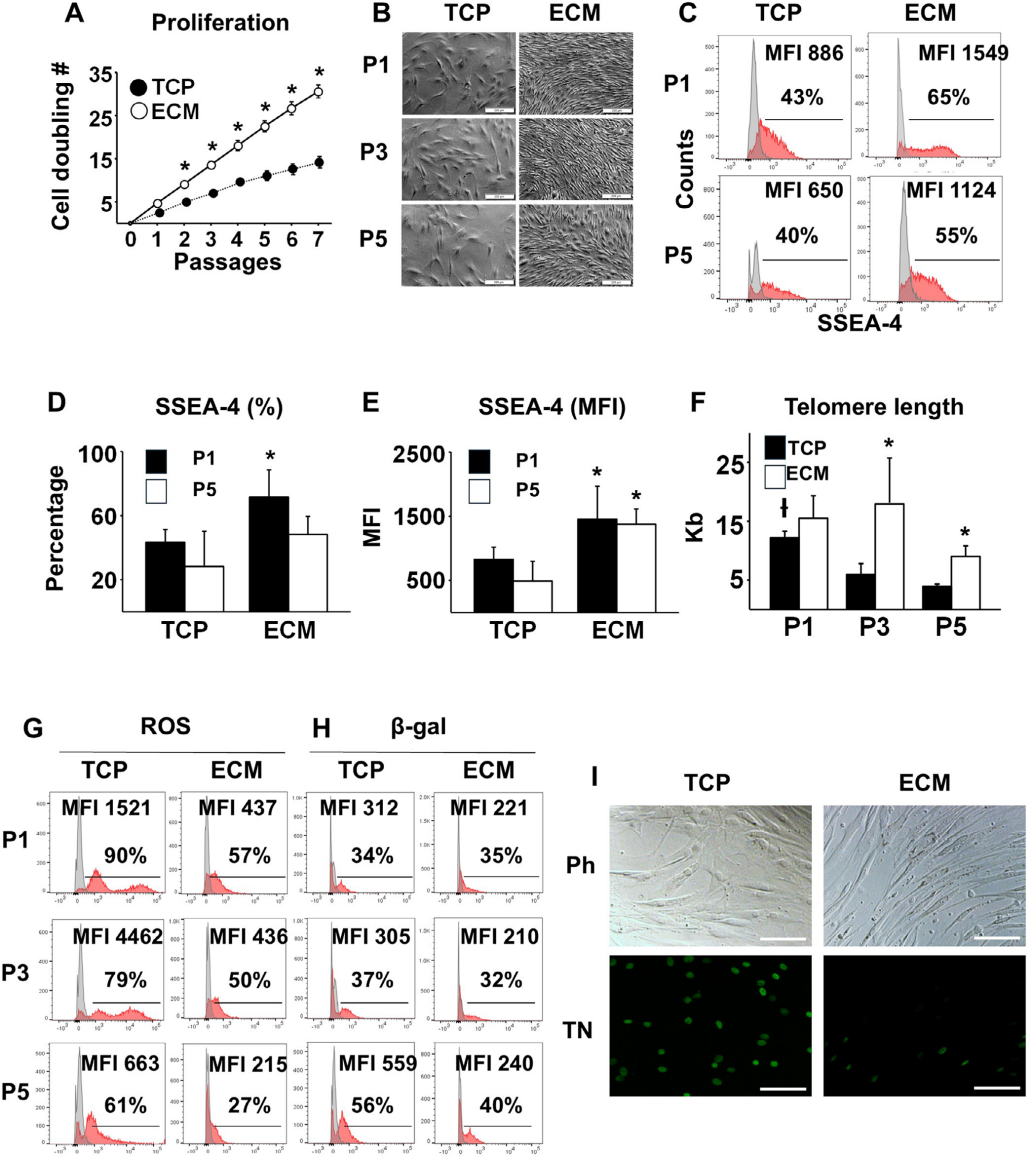
The quantity and quality of aging AD‐MSCs is improved with maintenance on ECM Plus (ECM) as compared to TCP. (A) Cell proliferation, based on the number of cell population doublings with each passage (7 days/passage), was enhanced with culture on ECM; maintenance on ECM for 7 passages resulted in ~1 × 10^6^‐fold greater number of cells than culture on TCP for the same number of passages. *p < 0.05 (*n* = 3), versus TCP at the same passage. (B) Cells sub‐cultured (7 days/passage) on TCP or ECM were viewed under phase contrast microscopy at the end of each passage (P1, P3, and P5) and photomicrographs prepared. Scale bar: 200 μm. (C) Flow cytometric analysis of SSEA‐4 expression was performed on cells after sub‐culture on TCP or ECM. P1 and P5: Passage 1 and 5, respectively; and MFI: Median fluorescence intensity. (D) Quantification of percent SSEA‐4 positive cells. *p < 0.05 (*n* = 3), versus TCP at the same passage. (E) Quantification of the mean fluorescence intensity (MFI) of SSEA‐4 positive cells. *p < 0.05 (*n* = 3), vs. TCP at the same passage. (F) Change in telomere length (Kb) with serial sub‐culture on TCP or ECM (P1, P3, and P5) was measured to assess cell quality. *p < 0.05 (*n* = 3), versus TCP at the same passage; and ^Ɨ^ *p* < 0.05 (*n* = 3), versus TCP at P3 and P5. (G) Flow cytometric analysis for production of reactive oxygen species (ROS), shown as a percentage (%) and MFI of positive cells, was performed on cells after sub‐culture (P1, P3, and P5) on TCP or ECM. (H) Flow cytometric analysis for β‐galactosidase (β‐gal) activity, shown as a percentage (%) and MFI of positive cells, was performed on cells after sub‐culture on TCP or ECM (P1, P3, and P5). (I) Apoptosis detected by TUNEL assay. Aging AD‐MSCs (P1) were cultured on TCP or ECM for 7 days and stained with TUNEL. Apoptotic cells (stained green) were more abundant on TCP (27% ± 9%) than ECM (6% ± 2%) and the increase on TCP was statistically significant (*p* < 0.01, *n* = 5). Ph: Phase contrast; TN: TUNEL‐fluorescence. Scale bar: 100 μm.

The cells maintained on ECM Plus had a stable doubling time of ~35 h, resulting in a steady increase in population doubling through 7 passages; in contrast, the cells maintained on TCP had a doubling time of ~92 h, resulting in a population doubling that plateaued after P3. After 7 passages, the number of cells maintained on ECM Plus was ~1 × 10^6^‐fold greater than on TCP (Figure 1A). Throughout each sub‐culture, aging AD‐MSCs on ECM Plus were evenly distributed across the cell culture surface, displaying a smaller size and more fibrillar morphology relative to cells on TCP (Figure 1B). Expansion on ECM Plus also conferred aging AD‐MSCs with increased expression of SSEA‐4 (Figure 1C–E), an early‐stage stem cell marker (Gang et al. 2007); cells cultured on ECM Plus versus TCP contained a higher percentage of SSEA‐4 positive cells that was significant at P1 (Figure 1D) and a higher median fluorescence intensity (MFI) that was significant at both P1 & P5 (Figure 1E). To investigate whether the enhanced proliferation of aging AD‐MSCs maintained on ECM Plus was associated with improved cell quality, we measured telomere length and found that it was preserved when AD‐MSCs were sub‐cultured on ECM Plus versus TCP (Figure 1F); this result was consistent with the improvement seen earlier with cell proliferation. Reactive oxygen species (ROS) and β‐galactosidase activity (β‐gal), a marker of senescence, were also significantly reduced when aging AD‐MSCs were sub‐cultured on ECM Plus versus TCP (Figure 1G,H). Furthermore, cells maintained on ECM Plus, as compared to TCP, displayed a marked decrease in cellular apoptosis (Figure 1I).

**FIGURE 2:**
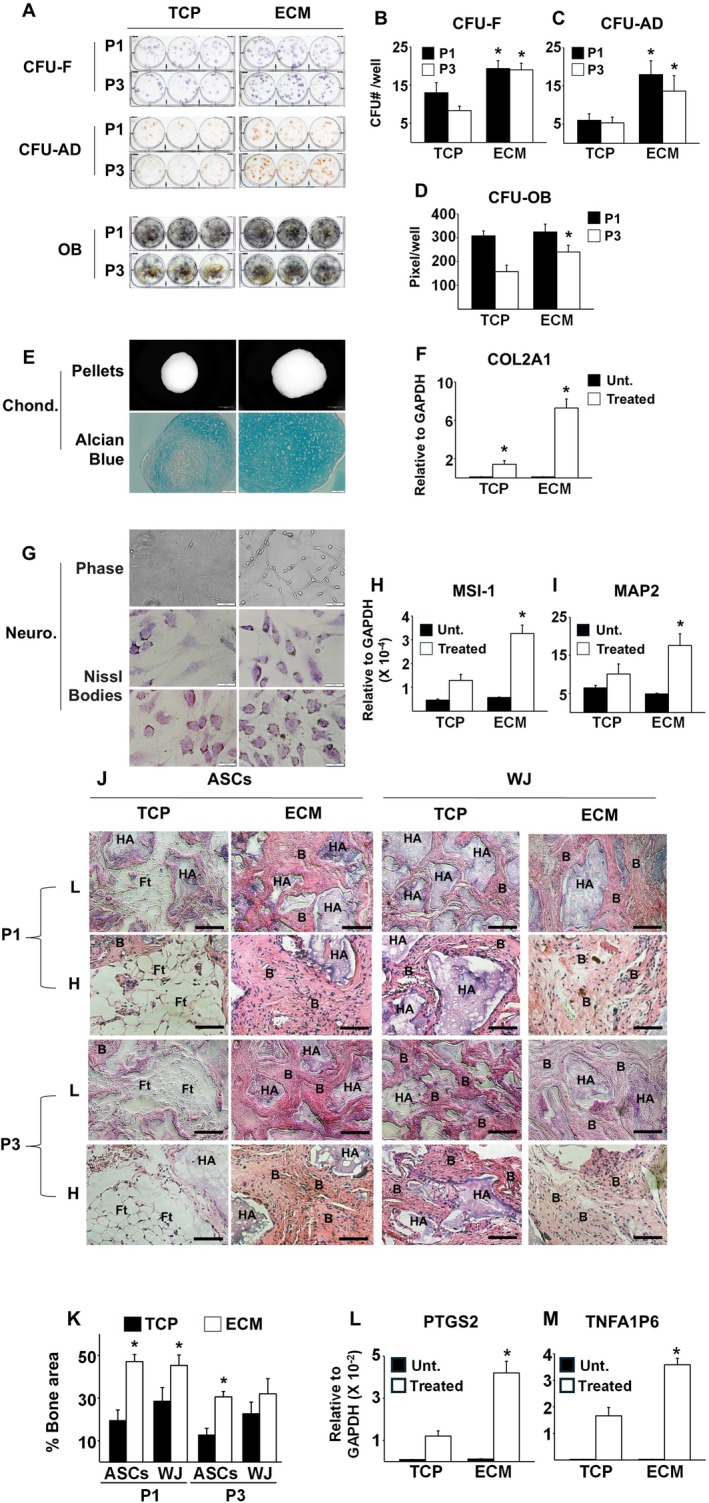
Differentiation capacity of aging AD‐MSCs is enhanced with maintenance on ECM Plus (ECM) as compared to TCP. (A) Formation of colony‐forming units (CFUs) was enhanced when cells were pre‐cultured on ECM as compared to TCP (P1 and P3). CFU‐fibroblasts (CFU‐F) were stained with crystal violet (blue); CFU‐adipocytes (CFU‐AD) were stained with Oil Red O (red); and CFU‐osteoblasts (CFU‐OB) were stained with von Kossa. (B–D) CFU quantification was performed by counting the number of colonies/well (CFU#/well) or pixels per 10 cm^2^ well. *p < 0.05 (*n* = 3), versus TCP at the same passage. (E, F) Generation of cartilage was enhanced when cells were pre‐cultured on ECM as compared to TCP. Transverse sections of cartilage pellets, stained with Alcian Blue, clearly demonstrated the presence of mucins and proteoglycans. In addition, the expression of type II collagen (COL2A1), a major collagenous protein of cartilage, was also significantly increased by pre‐culture on ECM. *p < 0.05 (*n* = 3), versus TCP group treated with chondrogenic media. (G–I) Neurogenesis was enhanced when the cells were pre‐cultured on ECM as compared to TCP. After overnight incubation in neurogenic induction media, a marked morphological difference between cells pre‐cultured on ECM versus TCP could be seen under phase contrast microscopy. Moreover, neuron‐specific Nissl body staining was observed and transcripts for neurogenic markers, musashi1 (Msi1) and microtubule associated protein 2 (MAP2) were increased. *p < 0.05 (*n* = 3), versus TCP group treated with neurogenic media. (J) In vivo bone formation capacity of cells pre‐cultured on ECM was enhanced compared to TCP (P1 or P3). Following each passage, aging AD‐MSCs (ASCs) or Wharton Jelly (WJ) cells pre‐cultured on TCP or ECM (1 × 10^6^ cells) were loaded onto HA/TCP particles and implanted subcutaneously into the dorsal surface of 10‐week‐old immunodeficient mice. After 8 weeks in vivo, three implants for each group were harvested for histological analysis. B, bone‐like regions; Ft, fat tissue; and HA, HA/TCP. Low (L) magnification: Scale bar: 200 μm. High (H) magnification: Scale bar: 100 μm. (K) Quantification of new bone formation (“% bone area”) was histomorphometrically determined using Image J analysis software. The data shown in the figure represents the mean and standard deviation that was calculated using 9 H&E sections (3 per implant, repeated 3 times to have 3 implants with cells from 3 different donors). *p < 0.05 (*n* = 9), versus TCP group. (L, M) Trophic factor expression by the cells was increased with maintenance on ECM as compared to TCP. To simulate an inflammatory environment, cells pre‐cultured on ECM or TCP were treated with TNF‐α (20 ng/mL) for 48 h and the expression of prostaglandin‐endoperoxide synthase 2 (PTGS2), which encodes the cyclooxygenase 2 (COX‐2) enzyme, and TNF‐α induced protein 6 (TNFA1P6) was measured by RT‐PCR. *p < 0.05 (*n* = 3), versus TCP group treated with TNF‐α.

We next investigated whether culture on ECM Plus also improved the differentiation capacity of aging AD‐MSCs. To limit the differential effects of the two cell culture surfaces on the assays, AD‐MSCs pre‐cultured on TCP or ECM Plus were subsequently plated onto the same culture substrate (TCP) at the same seeding density and induced by specific differentiation media. Initially, we compared the MSC frequency of cells pre‐cultured on TCP versus ECM Plus by measuring colony‐forming unit‐fibroblasts (CFU‐F). Clearly, aging AD‐MSCs pre‐cultured on ECM Plus contained an increased number of CFU‐F relative to TCP, suggesting an enrichment of MSCs with superior self‐replicative properties in this population (Figure 2A,B). Since not all CFU‐F are able to differentiate, we then treated CFU‐F with differentiation media to induce adipogenesis or osteoblastogenesis. The enriched CFUs also displayed enhanced differentiation potential, as cells pre‐cultured on ECM Plus produced a greater number of CFU‐adipocytes (CFU‐AD) (Figure 2A,C) and osteoblasts (OB) (Figure 2A,D). Furthermore, after 21 days in chondrogenic induction media, the cartilage pellets generated by AD‐MSCs pre‐cultured on ECM Plus were larger and contained more ECM and proteoglycans than those produced by cells pre‐cultured on TCP (Figure 2E). Consistently, aging AD‐MSCs pre‐cultured on ECM Plus had higher expression of COL2A1 relative to TCP (Figure 2F). Our findings also showed that pre‐culture on ECM Plus enhanced neurogenic differentiation of AD‐MSCs (Figure 2G). After overnight incubation in neurogenic induction media, there was a pronounced morphological difference between AD‐MSCs pre‐cultured on ECM Plus versus TCP. By the end of Day 10 in neurogenic media, AD‐MSCs pre‐cultured on ECM Plus expressed higher levels of musashi1 (Msi1) and microtubule associated protein 2 (MAP2), markers of neural progenitor cells, and formation of neuron‐specific Nissl bodies (Figure 2G–I).

To compare the bone‐forming capacity of early‐passaged (P1 or P3) aging AD‐MSCs with “youthful” WJ cells expanded on TCP or ECM Plus, we subcutaneously implanted an equivalent number of cells (1 × 10^6^), loaded onto HA/TCP particles, into immune‐compromised NIH‐bg‐nu‐xid mice. After 8 weeks, the implants were harvested for histological analysis. The amount of bone generated by aging AD‐MSCs pre‐cultured on TCP for P1 and P3 was about 26% and 15%, respectively, of the total ossicle area, which was also characterized by a large number of adipocytes (Figure 2J,K). In contrast, the implantation of AD‐MSCs pre‐cultured on ECM Plus for P1 and P3 generated about 47% and 31%, respectively, of the total ossicle area, which was ~2‐fold more than that produced by cells pre‐cultured on TCP. Although the amount of bone tissue generated by the aging AD‐MSCs maintained on ECM Plus decreased with increasing passage, the levels were always higher than that of cells maintained on TCP (Figure 2K). Interestingly, it was rare to find adipocytes in the ossicles formed by AD‐MSCs pre‐cultured on ECM Plus (Figure 2J). Although there was improvement in WJ cell bone formation capacity when pre‐cultured on ECM Plus (~47% vs. 30%, respectively) (Figure 2J,K), this effect was not as pronounced as that of aging AD‐MSCs. No bone formation was observed in the cell‐free scaffold implants (data not shown).

To assess the effect of cell culture substrate on trophic factor production, aging AD‐MSCs pre‐cultured on TCP or ECM Plus were treated with 20 ng/mL TNF‐α for 48 h to simulate a pathologically relevant inflammatory environment (Lee et al. 2017); the expression of prostaglandin‐endoperoxide synthase 2 (*PTGS2*), which encodes cyclooxygenase 2 (COX‐2), and TNF‐α induced protein 6 (*TNFA1P6*) was measured to represent an MSC in vivo anti‐inflammatory response. The results showed that AD‐MSCs pre‐cultured on ECM Plus expressed much higher levels of these soluble anti‐inflammatory factors in response to TNF‐α than cells pre‐cultured on TCP (Figure 2L,M). Taken together, these results demonstrate that maintenance of AD‐MSCs on ECM Plus remarkably restores the ability of the cells to self‐renew, undergo differentiation into multiple cell lineages, including osteogenesis, in vivo, and produce trophic factors.

### 3.2 Maintenance of Aging AD‐MSCs on ECM Plus Promotes Mitochondrial Respiratory Chain Super‐Complex Assembly and Enhances Coupling Efficiency

**FIGURE 3:**
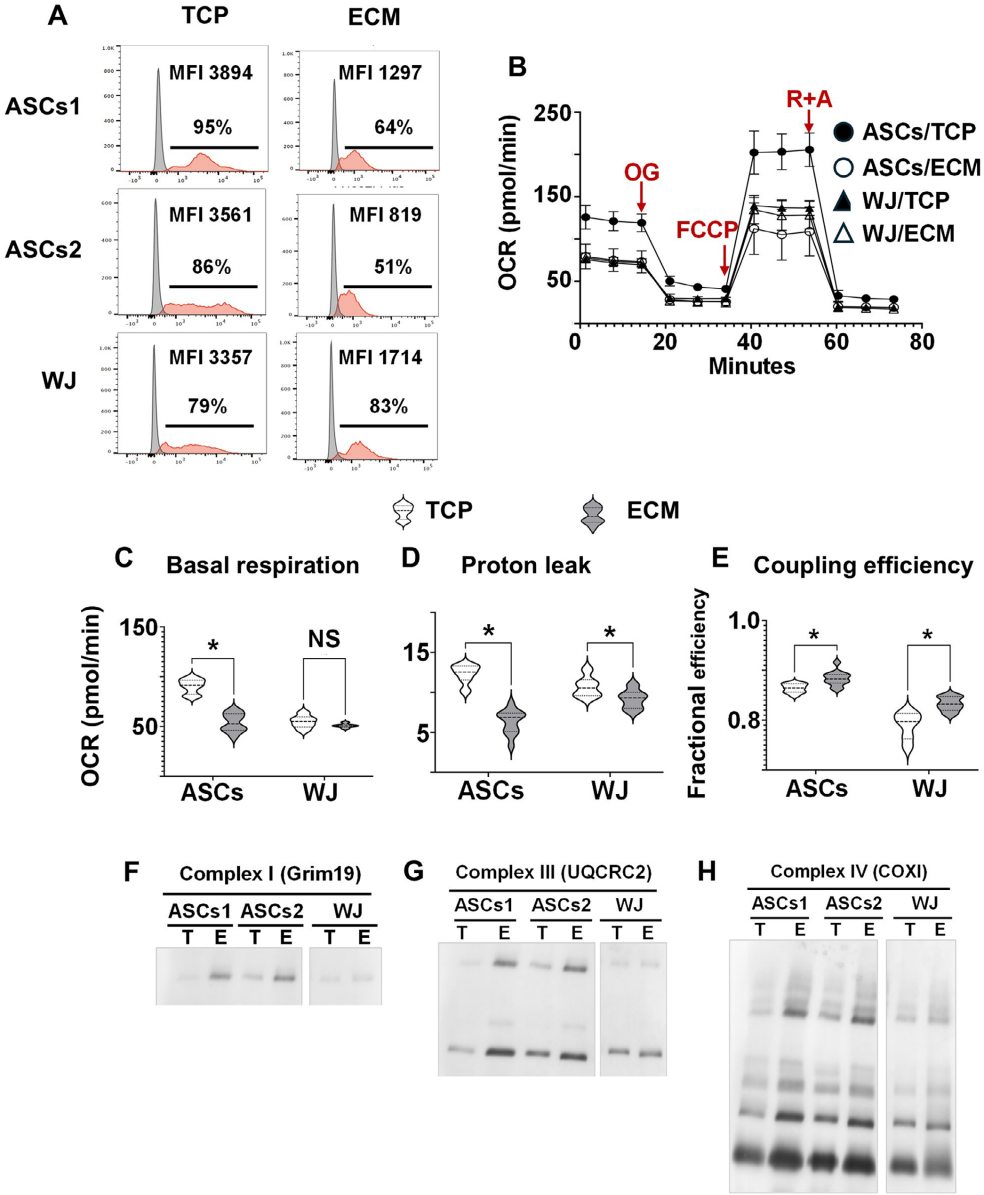
Culture of aging AD‐MSCs on ECM Plus (ECM), but not TCP, produces less ROS by promoting respiratory chain super‐complex assembly and enhanced coupling efficiency. (A) ROS production by aging AD‐MSCs (ASCs, P2, or P3) or WJ cells (< P5) pre‐cultured on ECM or TCP was measured by flow cytometry. ROS production is shown as both the percent positive cells in the population and mean fluorescence intensity (MFI). Two separate donors provided the aging AD‐MSCs (ASCs1 and ASCs2), TCP: 91% ± 9% versus ECM: 58% ± 12% (*p* < 0.05, *n* = 6); TCP: 3730 ± 340 MFI versus ECM: 1150 ± 459 MFI (*p* < 0.05, *n* = 6); WJ cells were used as youthful (young) control cells, TCP: 75% ± 7% vs. ECM: 80% ± 5% (not significantly different); TCP: 3542 ± 256 MFI vs. ECM:1752 ± 270 MFI (*p* < 0.05, *n* = 3). (B–E) Seahorse analysis. Cells pre‐cultured on TCP (ASCs/TCP or WJ/TCP) or ECM (ASCs/ECM or WJ/ECM) were plated at 40,000 cells per well in an XF96 cell culture microplate (Seahorse Bioscience) for the Seahorse Mito Stress Test, as described in the Methods. Oxygen consumption rate (OCR) was measured at baseline and after sequential administration of oligomycin (OG) (2.0 μM), FCCP (1.0 μM) and rotenone (0.6 μM)/antimycin A (*R* + A) (0.5 μM). Based on the results, indices of mitochondrial function were calculated and included basal respiration (C), proton leak (D), and coupling efficiency (i.e., oxidative phosphorylation [OXPHOS]) (E). *p < 0.05 (*n* = 3), for ECM vs. TCP pre‐culture. (F–H) Blue Native Gel (BNG) analysis was used to investigate assembly of the respiratory super‐complex. Mitochondrial protein was extracted from ASC and WJ cells pre‐cultured on ECM (E) or TCP (T), separated using gel electrophoresis, and the protein complexes detected by western blot (Wb) using antibodies against representative subunits of complex I, III, and IV.

In separate experiments, using cells from different donors, we compared ROS production between aging AD‐MSCs cultured on TCP versus ECM Plus and used WJ cells as “youthful” control cells. The results showed that AD‐MSCs cultured on ECM Plus displayed a more “youthful” phenotype, like that observed with WJ cells, characterized by a significantly lower MFI for ROS staining relative to TCP (Figure 3A) suggesting that culture on ECM Plus promoted the growth of cells with superior antioxidant properties.

To determine the underlying mechanism responsible for the effect of ECM Plus on ROS production, we investigated cellular mitochondrial respiration on the two surfaces by measuring oxygen consumption rate (OCR) during a Seahorse XF Mito Stress test, which was comprised of three phases: (i) oligomycin (OG) to block ATP synthase, (ii) carbonyl cyanide‐4 (trifluoromethoxy) phenylhydrazone (FCCP) to disrupt mitochondrial membrane potential, and (iii) rotenone/antimycin A (*R*+A) to block mitochondrial respiration (Figure 3B). The results showed that prior to OG treatment, AD‐MSCs maintained on ECM Plus had a comparable basal respiration rate to the “youthful” WJ cells maintained on either TCP or ECM Plus, which was significantly lower than that of AD‐MSCs cultured on TCP (Figure 3C). Interestingly, AD‐MSCs as well as WJ cells cultured on ECM Plus displayed a significant decrease in proton leakage (i.e., protons that cross the mitochondrial membrane but do not contribute to ATP synthesis), as compared to cells cultured on TCP (Figure 3D). The reduction in proton leakage was accompanied by an increase in respiratory coupling efficiency, computed as the ratio of ATP‐linked respiration to basal respiration (Figure 3E). These results suggested that the restorative effects of culture on ECM Plus were likely achieved by suppressing proton leakage, leading to an enhancement in energy efficiency and lower metabolic burden.

To further reveal the molecular mechanism underlying the improvement in oxidative phosphorylation (OXPHOS) coupling efficiency associated with culture on ECM Plus, we conducted a Blue Native Gel (BNG) analysis to investigate the assembly of the mitochondrial respiratory chain, focusing on respiratory supercomplex assembly (Qin et al. 2024). The results showed that the respiratory chain supercomplex (i.e., respirasome), containing complexes I, III, and IV, was clearly enhanced by culturing the aging AD‐MSCs on ECM Plus (Figure 3F–H), which was not significantly observed with the WJ cells. These results suggested that ECM Plus promoted the assembly of the electron transport chain supercomplex in aging cells, leading to enhanced coupling efficiency and suppressed production of ROS.

### 3.3 Single‐Cell RNA‐Seq Analysis Reveals a Unique Gene Expression Profile of Aging AD‐MSCs Post‐Rejuvenation by Culture on ECM Plus Versus TCP

**FIGURE 4:**
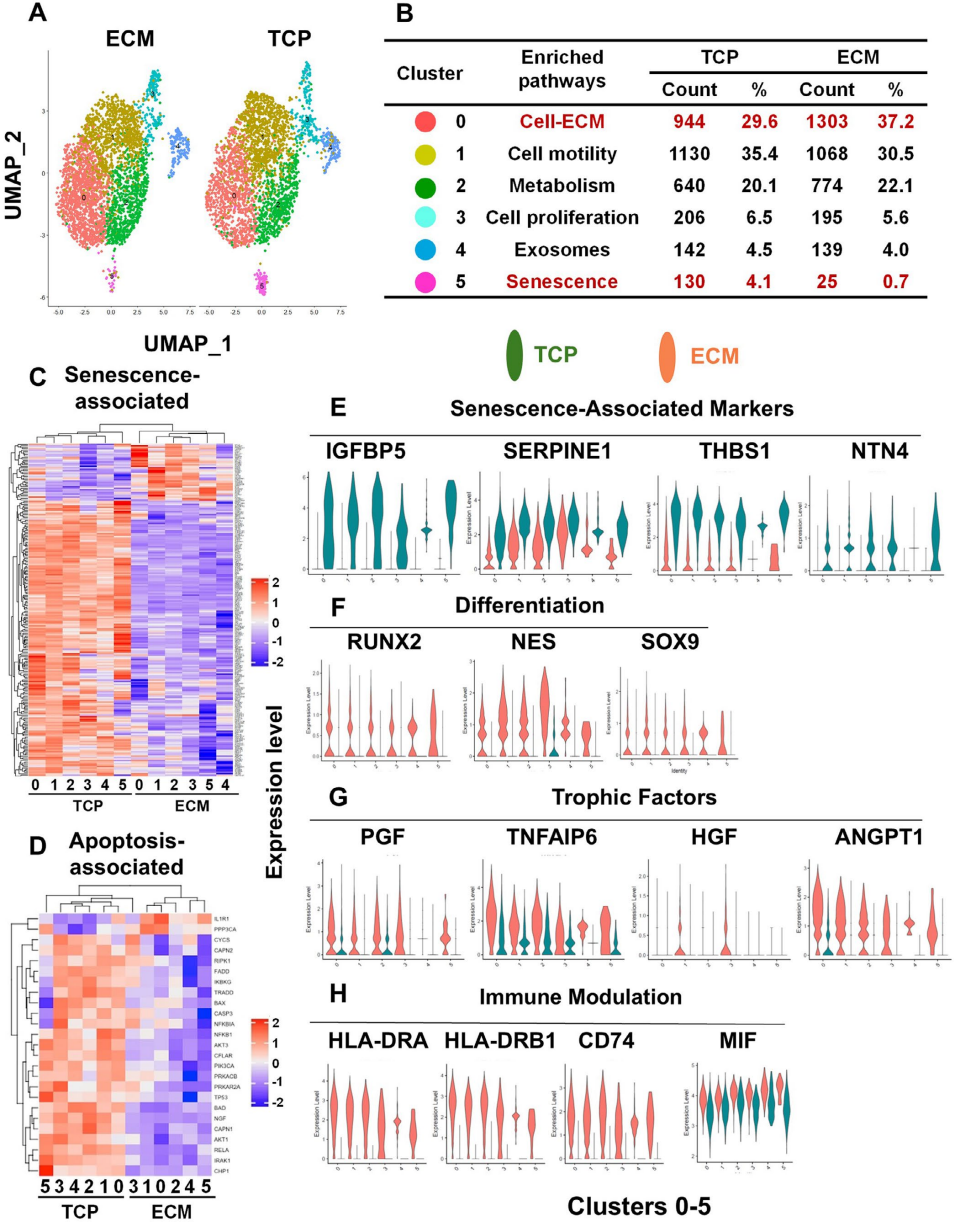
Comparative single cell transcriptomic analysis reveals that the culture of aging AD‐MSCs on ECM Plus (ECM) produces a unique gene expression profile, including the downregulation of pro‐senescent signaling. (A) UMAP (uniform manifold approximation and projection) diagram for cells pre‐cultured on ECM Plus (ECM) versus TCP in regular growth media. (B) Six subpopulations or clusters were identified based on similar functionality or pathway enrichment. Cluster 0 (Cell‐ECM) and cluster 5 (Senescence) were highlighted due to the major shift in cell sub‐population composition between ECM and TCP. (C) Heatmap of senescence‐associated genes within DEGs (differentially expressed genes) by comparing cells cultured on TCP to ECM. A list of the genes displayed in the figure can be found in Table S1 in the Supporting Information. (D) Heatmap of apoptosis‐associated genes within DEGs by comparing cells cultured on TCP to ECM; the figure is enlarged in the Supporting Information (Figure S1) to show the individual genes. (E–H) A small number of DEGs, from 4 different categories (i.e., senescence‐associated markers, differentiation, trophic factors, and immune modulation), were selected as biomarkers of MSC quality. Identity 0–5 (along the *x*‐axis) refers to the cluster numbers in Figure 4B; pink‐colored violin plots represent the subpopulations cultured on ECM, while the dark green colored violin plots represent the subpopulations cultured on TCP.

To identify and characterize the different subpopulations of AD‐MSCs present after culture on ECM Plus versus TCP and further understand the underlying regulatory mechanisms involved, we performed single‐cell RNA‐seq analysis. By use of the Enrichr pathway analysis tool, we determined that the cultured cells were divided into 6 clusters or subpopulations, labeled C0–C5 (Figure 4A), and each cluster was enriched for a specific pathway involving a different biological function (Figure 4B). The clusters displaying the most notable differences for aging AD‐MSCs maintained on TCP versus ECM Plus were: C0: cell‐ECM interactions (29.6% vs. 37.2%, respectively) and C5: senescence (4.1% vs. 0.7%, respectively). These results suggested that culture on ECM Plus had the most profound effect on activating cell‐ECM interactions and inhibiting cellular senescence.

To further refine our focus on senescence as well as apoptosis‐associated differentially expressed genes (DEGs), hierarchical clustering revealed an attenuation in expression of the senescence‐associated secretory phenotype (SASP) in aging AD‐MSCs maintained on ECM Plus compared to cells on TCP (Figure 4C). In addition, the expression of apoptosis‐associated genes was also decreased when aging AD‐MSCs were maintained on ECM Plus versus TCP (Figure 4D). The identity of the genes shown in the heatmaps (Figure 4C,D) is listed in the Supporting Information section (Table S1, Figure S1). Overall, the results showed a consistent and relatively intense suppression of both cellular senescence and apoptosis, explaining the observations that we demonstrated previously with culture on ECM Plus (Figure 1H,I).

Next, we selected a small number of genes that displayed an extremely large differential expression profile between cells cultured on TCP and ECM Plus in the senescence‐associated, differentiation, trophic factors, & immune modulation categories (Figure 4E–H) to be validated by RT‐PCR (data not shown) or Western blot (Wb) analysis (Figure 6). Overall, these gene profiles supported and complemented our earlier observations showing that aging AD‐MSCs maintained on ECM Plus displayed a restoration in cell proliferation and suppression of SASP (Figure 4E); a simultaneous upregulation in the expression of *Runx2*, *NES*, and *SOX9*, indicating multi‐lineage differentiation potential to osteoblasts, neuron‐like cells, and cartilage, respectively (Figure 4F); and upregulation of various trophic factors that support an enhanced anti‐inflammatory response (Figure 4G). More interestingly, single‐cell RNA‐seq analysis suggested that ECM Plus strongly promoted the expression of the HLA‐DR complex and CD74, markers that had almost no expression on TCP (Figure 4H). Because CD74 is a component of the HLA‐DR complex and also a receptor for macrophage migration inhibitory factor (MIF), which activates several important cell signaling pathways, including the PI3K/Akt pathway to promote cell survival (Lue et al. 2007), we examined MIF expression and found no significant difference in cells cultured on TCP versus ECM Plus(Figure 4H).

### 3.4 Aging AD‐MSCs Cultured on ECM Plus Express Cell Surface HLA‐DR and Stimulate T Cell Proliferation

**FIGURE 5:**
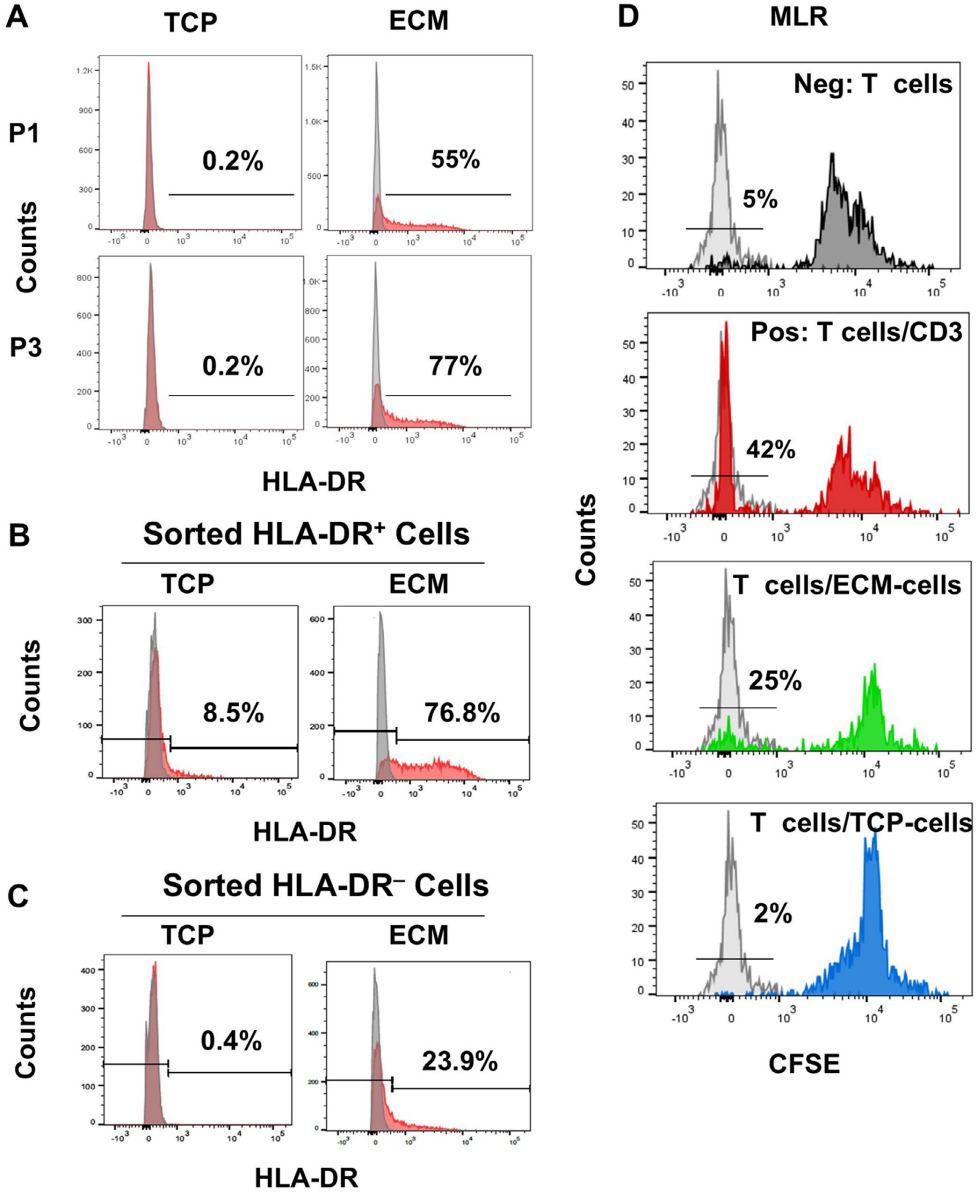
Expression of HLA‐DR on the aging AD‐MSC surface is only observed with culture on ECM Plus (ECM) and stimulates T‐cell proliferation. (A) Flow cytometric analysis of HLA‐DR expression by cells sub‐cultured on TCP or ECM (P1 and P3). (B, C) Aging AD‐MSCs were pre‐cultured on ECM, fractionated (sorted) into HLA‐DR positive (B) and HLA‐DR negative (C) sub‐populations by flow cytometry, and then cultured on TCP or ECM for 7 days. At the end of culture, the percentage of HLA‐DR positive and negative cells was determined by flow cytometric analysis. (D) A mixed lymphocyte reaction (MLR) assay. Aging AD‐MSCs pre‐cultured on TCP (TCP‐cells) or ECM (ECM‐cells) were cocultured with PBMCs pre‐labeled with CFSE. After 5 days of culture, dividing T‐cells were identified by CD4 positive cells with decreased AF488 (CFSE) intensity using flow cytometry.

Single‐cell RNA‐seq analysis suggested that culture of aging AD‐MSCs on ECM Plus up‐regulated the expression of HLA‐DR. This was further confirmed by flow cytometry showing that approximately 50%–80% of the cells were HLA‐DR^+^ (Figure 5A). However, CD80 and CD86, co‐stimulatory molecules involved in antigen presentation (Schwartz 1991), were not detected on the HLA‐DR^+^ cells (data not shown). Moreover, when aging AD‐MSCs (pre‐cultured on ECM Plus) were fractionated into HLA‐DR^+^ and HLA‐DR^−^ cells by fluorescence‐activated cell sorting (FACS) and then cultured on TCP or ECM Plus for 7 days, the percentage of HLA‐DR^+^ cells after culture on TCP decreased to ~8% while it remained at ~80% when they were cultured on ECM Plus(Figure 5B). In addition, HLA‐DR^−^ cells cultured on ECM Plus for 7 days increased to ~24% positive cells, but this did not occur in the cells cultured on TCP (Figure 5C). These data clearly indicated that upregulation of HLA‐DR expression by the cells was directly associated with culture on ECM Plus and interaction with the microenvironmental cues embedded in the matrix.

Next, we found that AD‐MSCs precultured on ECM Plus (ECM‐cells) stimulated T cell proliferation in a mixed lymphocyte reaction (MLR) assay (Figure 5D). In contrast, the extent of T cell proliferation was significantly less when the AD‐MSCs had been precultured on TCP (TCP‐cells), which is consistent with published results.

### 3.5 Aging AD‐MSCs Display Restored Multi‐Lineage Differentiation Capacity After Culture on ECM Plus

**FIGURE 6:**
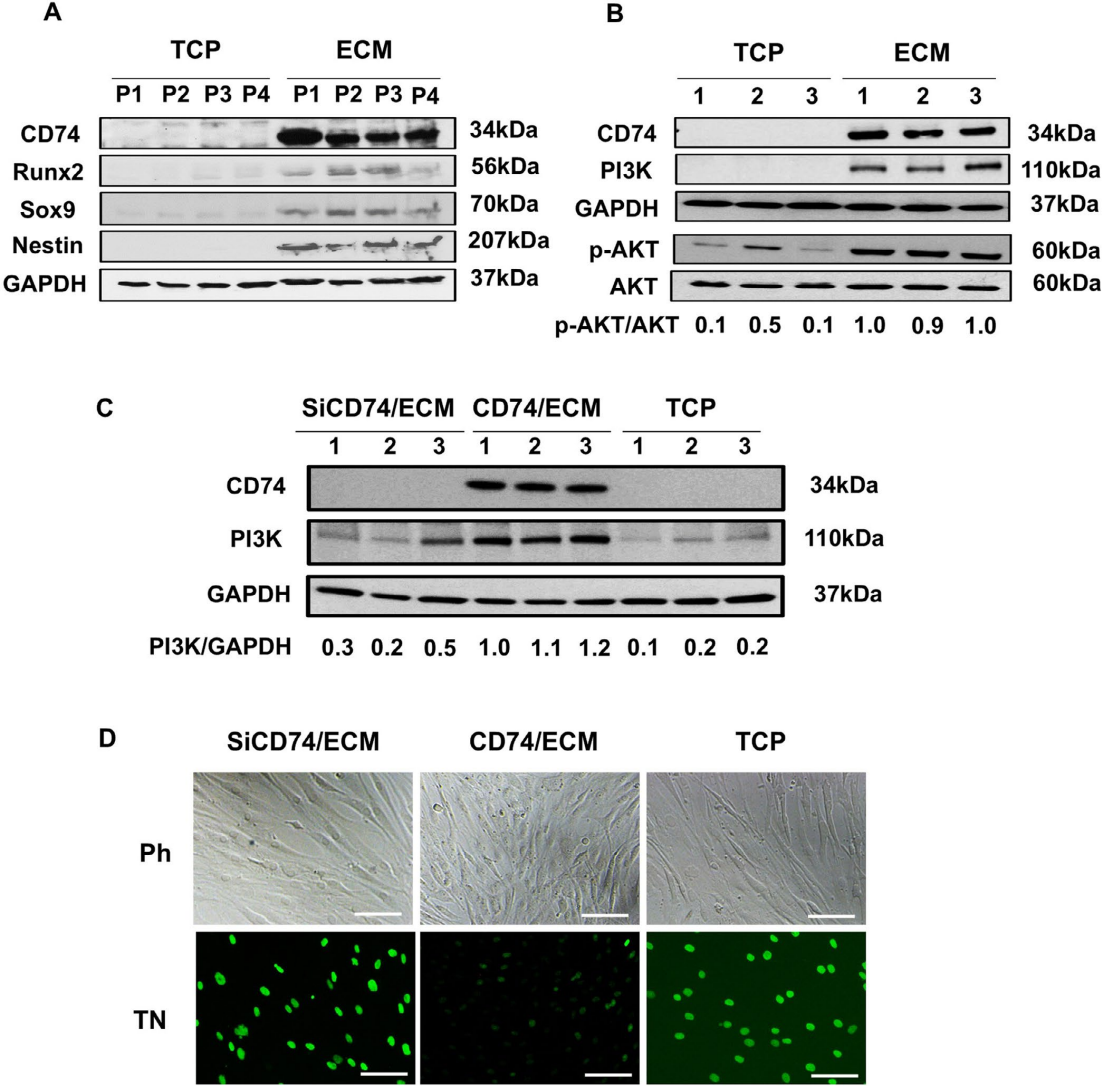
Western blot (Wb) analysis confirms the up‐regulation of CD74 and other key differentiation markers and reveals that CD74 is essential for the induction of PI3K signaling and prevention of cell death (i.e., apoptosis). (A) CD74, Runx2, Sox9, and Nestin proteins were produced by cells cultured on ECM Plus (ECM), as compared to TCP, through four serial passages (P1 to P4) in regular growth media. (B) In separate experiments, extracts of cells (from 3 different donors, > 65 years old) that had been pre‐cultured on ECM Plus (ECM), as compared to TCP, were analyzed by Wb analysis for the presence of CD74 and PI3K/AKT proteins. (C) Wb analysis for CD74 and PI3K protein in extracts of cells cultured on ECM Plus (ECM) and treated with siRNA to silence the expression of CD74 or scrambled siRNA (a negative control), as well as cells cultured on TCP (SiCD74/ECM vs. CD74/ECM vs. TCP, respectively). CD74 protein was undetected in CD74 silenced cells cultured on ECM or the naïve cells cultured on TCP, along with less PI3K production, as compared to cells treated with scrambled siRNA and maintained on ECM. Whole blots of panels A–C can be found in the Supporting Information section (Figure S2). (D) TUNEL assay for apoptosis was conducted on the above cells. Calculated percent TUNEL positive for each treatment group: SiCD74/ECM: 34% ± 8%; CD74/ECM: 12% ± 4%; TCP: 31% ± 5%; *p* < 0.01 (*n* = 5), CD74/ECM versus SiCD74/ECM or TCP with a one‐way ANOVA. Ph, phase contrast; TN, TUNEL‐fluorescence. Scale bar: 100 μm.

To confirm the single‐cell RNA‐seq results showing that culture on ECM Plus up‐regulated expression of Runx2, Sox9, and Nestin at the protein level, in separate experiments using cells from different donors, we serially subcultured (7 days/passage) aging AD‐MSCs on TCP or ECM Plus and then prepared protein extracts of the cells for Wb analysis. The results revealed that cells cultured on ECM Plus, as compared to TCP, displayed a remarkable increase in CD74 protein, as well as Runx2, Sox9, and Nestin protein, through four passages (Figure 6A).

### 3.6 CD74 Is Essential for Protecting Cells From Apoptosis via PI3K Signaling

Since CD74 is an HLA‐DR (class II) chaperone, as well as a receptor for MIF (Leng et al. 2003), we treated cells during culture on TCP or ECM Plus with varying concentrations of MIF. Unexpectedly, we failed to detect any response to MIF treatment, based on downstream signaling molecules (data not shown). However, there was a strong correlation between CD74 production and increased PI3K, subsequently leading to higher levels of p‐AKT, when aging AD‐MSCs were maintained on ECM Plus as compared to TCP (Figure 6B). To further demonstrate that the presence of CD74 was associated with the induction of PI3K, we silenced CD74 expression when the cells were cultured on ECM Plus (Figure 6C). The results showed that CD74 expression was successfully silenced in cells cultured on ECM Plus and that the silenced cells also displayed a dramatic reduction in PI3K, which was very similar to that of cells cultured on TCP. More importantly, the silencing of CD74 expression in cells cultured on ECM Plus resulted in the loss of protection from cell death (apoptosis), producing a high number of apoptotic cells, which was very similar to that of cells cultured on TCP (Figure 6D).

## 4 Discussion

The present study showed that the maintenance of aging AD‐MSCs on ECM Plus promoted cell replication with a short and stable doubling time of ~35 h, as compared to TCP with a doubling time of ~92 h; thus, subculture on ECM Plus for 7 passages amplified the number of cells ~1 × 10^6^ times more than maintenance on TCP (Figure 1A). Aging AD‐MSCs cultured on ECM Plus were small in size, enriched in early‐stage MSCs (i.e., SSEA‐4 positive), maintained their telomere length, and displayed a reduction in cellular senescence and apoptosis (Figure 1B–J), suggesting that culture of ECM Plus increased both the quantity and quality of aging AD‐MSCs. These results were confirmed by functional assays, where aging AD‐MSCs pre‐cultured on ECM Plus displayed a significant improvement in their multi‐lineage differentiation capacity and trophic factor production (Figure 2). In the present study, we also compared the ability of aging AD‐MSCs pre‐cultured on ECM Plus to form new bone in vivo with that of cells pre‐cultured on TCP and “youthful” WJ cells pre‐cultured on both substrates. The results showed that aging AD‐MSCs expanded on ECM Plus produced more than twice the amount of bone‐like tissue than the same number of cells expanded for 1 and 3 passages on TCP; moreover, the amount of bone formed by the aging AD‐MSCs on ECM Plus was comparable to that of WJ cells cultured under the same conditions (Figure 2J,K). Interestingly, we also found that implantation of AD‐MSCs pre‐cultured on TCP mainly formed adipose tissue, which was not seen when these cells were pre‐cultured on ECM Plus. This observation supports earlier findings showing that AD‐MSCs may naturally favor adipogenesis (Hayashi et al. 2008) and suggests that stem cell fate can be guided to a desired cell lineage by exposure to a tissue‐specific microenvironment (Marinkovic et al. 2016).

Since culture on ECM Plus significantly downregulated ROS production in aging AD‐MSCs, this suggested that this cell culture substrate might regulate cell respiration. Indeed, the data showed that the age‐related decline in mitochondrial respiration was attenuated by maintenance on ECM Plus—by preserving the mitochondrial respirasome, while culture on ECM Plus promoted efficient production of ATP, which reduced the production of ROS and mitigated oxidative damage to mitochondrial proteins, membranes, and DNA (Figure 3).

The molecular mechanism underlying the rescue of aging AD‐MSCs by culture on ECM Plus was investigated by single‐cell RNA‐seq analysis. The results showed that the cells were clustered into 6 subpopulations, based on their unique gene expression profiles (Figure 4A). The cluster showing the greatest difference in cellular composition for aging AD‐MSCs maintained on ECM Plus and TCP was cluster 5, senescence and apoptosis (Figure 4B) and heatmaps of this cluster revealed a down‐regulation of these genes with culture on ECM Plus (Figure 4C,D). A list of the individual genes and fold changes can be found in the Supporting Information section (Table S1, Figure S1). We selected some of these genes, such as *IGFBP5*, *SERPINE1*, *THBS1*, and *NTN4* (Figure 4E), based on their association with senescence in MSCs (Medeiros Tavares Marques et al. 2017), and confirmed the results using RT‐PCR. Aging AD‐MSCs cultured on ECM Plus also displayed an increase in simultaneous expression of *Runx2*, *NES*, and *SOX9* (Figure 4F), which was further confirmed at the protein level (Figure 6A). These findings clearly illustrate that multipotent differentiation capacity (i.e., osteoblastogenesis, neurogenesis, and chondrogenesis, respectively) was restored by maintenance, as well as serial subculture, on ECM Plus.

Surprisingly, single‐cell RNA‐seq analysis, as well as confirmation by flow cytometry, revealed that culture on ECM Plus promoted the expression of HLA‐DR (Figure 5A). Further, we observed that this phenomenon was specific to ECM Plus, as purified HLA‐DR^+^ cells rapidly lost their expression when transferred back to TCP and, conversely, purified HLA‐DR^−^ cells partially acquired expression once they were cultured on ECM Plus (Figure 5B,C). These data demonstrated that the up‐regulation of HLA‐DR expression was directly associated with culture on ECM Plus. Our results challenge the long‐held view that MSCs do not express HLA‐DR and strongly suggest that failure to express HLA‐DR is due to culture on an artificial substrate (i.e., TCP). Since the matrix proteins found in ECM Plus have been synthesized by human fetal cells and consist of ~39% collagens and ~49% glycoproteins (Block et al. 2020), this cell culture system better simulates the physiological microenvironment and cell behavior in vivo; thus, our findings imply that transplanted MSCs in vivo are likely to normally express HLA‐DR and explain rare reports suggesting that new tissue formation is directly generated by transplanted allogenic MSCs. Our findings support the notion that MHC class II expression by MSCs can be influenced by many factors (e.g., cell source, donor status, culture conditions, & host status) that lead to immunogenicity (van Megen et al. 2019; Chen et al. 2022). Huang et al. reported that allogeneic MSCs were eliminated by Week 5 after implantation in infarcted rat myocardia (Huang et al. 2010). In the present study, we also found that CD74 expression (at both the mRNA and protein levels) was upregulated along with HLA‐DR when aging AD‐MSCs were maintained on ECM Plus (Figures 4H and 6). As an HLA‐DR chaperone, CD74 in the endoplasmic reticulum (ER) is required for antigen processing and assists in the assembly and trafficking of MHC class II complexes (Schroder 2016). While HLA‐DR and CD74 expression did not correlate with the upregulation of CD80 and CD86 (T‐cell co‐stimulatory markers) (data not shown), aging AD‐MSCs cultured on ECM Plus gained the ability to process antigens and stimulate T cell proliferation (Figure 5D). Moreover, CD74 has been proposed as a marker which distinguishes BM‐MSCs from fibroblasts (Ishii et al. 2005), implying better retention of stem cell properties when MSCs are cultured on ECM Plus versus TCP.

Although CD74 is a receptor for MIF, independent of MHC class II (Leng et al. 2003), a downstream response to MIF treatment was not detected in our study. Interestingly, our results showed that cells maintained on ECM Plus, but not TCP, displayed an increase in PI3K/AKT signaling in the absence of MIF (Figure 6B). The PI3K/AKT pathway has been shown to play a crucial role in promoting cell survival and inhibiting programmed cell death (Hossini et al. 2016; Gong et al. 2020; Wang et al. 2023). To further determine whether the presence of CD74 is essential for the induction of PI3K signaling, we silenced CD74 expression in cells when maintained on ECM Plus by siRNA treatment and found that the CD74‐silenced cells showed a remarkable decrease in PI3K (Figure 6C) and a dramatic increase in apoptosis (Figure 6D). Here we report for the first time a novel role for intracellular CD74 in anti‐apoptosis via PI3K signaling, which was up‐regulated in AD‐MSCs cultured on ECM Plus. In the present study, we employed ECM Plus that is enriched in collagen XVIII, agrin, and perlecan (Block et al. 2020). These ECM proteins belong to the heparan sulfate proteoglycan (HSPG) family that bind a variety of ligands (e.g., growth factors, cytokines, chemokines, enzymes and ECM proteins) and play important roles in cell attachment, motility, growth, and preventing cellular senescence (Jung and Oh 2016; Sarrazin et al. 2011). Future studies will aim to further identify the key matrix component(s)/proteins that drive HLA‐DR and CD74 expression, as well as activation of the PI3K/AKT pathway, by silencing or over‐expressing each candidate protein during production of ECM Plus, as previously described (Marinkovic et al. 2022). Overall, the present study has prepared a solid foundation for initiating the development of a defined ECM culture system, based on using purified key ECM proteins or combinations of recombinant molecules, which will improve its reproducibility and scalability for use in clinical applications.

A limitation of the present study is that ideally old‐ECM (not TCP) would have been used to compare the growth of aging AD‐MSCs on ECM Plus. However, it is impossible to prepare an “old”‐ECM that is made by cells from the same tissue source (i.e., human amniotic fluid‐derived pluripotent stem cells). Moreover, it would be inappropriate to compare “old” BM‐ECM with ECM Plus because they are made by cells from different tissues. Previously, the protein composition of ECM Plus has been shown to be quite different from BM‐ECM and is superior in its ability to maintain hiPSCs (Block et al. 2020). Moreover, we have demonstrated that ECM plays a tissue‐specific role in controlling the fate of MSCs (Marinkovic et al. 2016). With these considerations in mind, we decided to compare cell behavior on ECM Plus with a standard TCP culture surface. A second limitation is that, theoretically, we should have used AD‐MSCs obtained from young donors as youthful controls for aging AD‐MSCs. However, there is an extensive body of scientific evidence, as well as our own experience, showing that the properties of AD‐MSCs from young donors are highly variable due to differences in gender, body mass index, donor collection site (e.g., abdomen, waist), and preparation method (Tsekouras et al. 2017; Gentile et al. 2019). In contrast, WJ cells consistently display a youthful phenotype and were used in the present study as a surrogate for “young” MSCs. However, we noticed that they showed less improvement in properties with culture on ECM Plus than AD‐MSCs. Our explanation for this is that WJ cells are considered fetal stem cells, and their self‐renewal and differentiation capacity are far superior to adult MSCs. Especially with early‐passage WJ cells, data from our lab has shown that these cells are less influenced by culture on ECM as compared to adult BM‐ or adipose‐derived MSCs.

## 5 Conclusion

This study demonstrated that aging AD‐MSCs could be rescued by culture on a native ECM synthesized by human AFCs (ECM Plus). The potential mechanisms were associated with a remarkable up‐regulation of intracellular CD74 in cells maintained on ECM Plus over TCP, which subsequently triggered activation of the PI3K pathway as a key modulator of cell survival and suppression of cellular senescence and apoptosis. Moreover, the present study clearly revealed that AD‐MSCs maintained on ECM Plus increased their expression of HLA‐DR which stimulated T cell activity. These findings challenge the dogma that MSCs are “immune evasive” or “immune privileged”, which has served as the foundation for a “one size fits all” and “off the shelf” approach for allogeneic MSC‐based therapies. Taken together, improvements in the quantity and quality of autologous MSCs for personal stem cell banks may result in a new paradigm for treating age‐related diseases; serial administration of rejuvenated autologous MSCs may not only replace aged MSCs, but also gradually reverse the aged microenvironment.

## Author Contributions

**Aaron O. Gonzalez:** collection and/or assembly of data, data analysis and interpretation, preparing the first draft of the manuscript. **Parveez A. Abdul Azees** and **Jerry P. Chen:** collection and/or assembly of data, specifically measurement of apoptosis in cells before and after silencing of CD74. **Brian Cao**, **Ting Liang**, **Peiqing Hu**, and **Yidong Bai:** design of studies involving the measurement of cellular mitochondrial respiration. **Milos Marinkovic**, **Chih‐Ko Yeh**, and **David D. Dean:** data analysis and interpretation, administrative support; manuscript writing. **Xiao‐Dong Chen:** conception and design of the study; financial support; data analysis and interpretation; manuscript writing; final approval of manuscript. All authors have reviewed and approved the final manuscript.

## Conflicts of Interest

Dr. Chen is a Board member and shareholder in StemBioSys Inc. (San Antonio, TX). All other authors have no financial or competing interests to declare.

## Supporting information


Appendix S1.

**Figure S1.** An enlarged version of Figure 4D which displays up‐ or down‐ regulated apoptosis‐associated genes expressed by cells maintained on TCP versus ECM.

**Figure S2.** Entire Wb blots used to create the blots shown in Figure 6A–C. The original blots showed some non‐specific bands and background. The indicated bands were identified by their molecular weight.

**Table S1.** Senescence‐associated genes up‐regulated by cell culture on TCP or ECM. The senescence‐associated genes were identified in cluster 5 (Figures 4B,C) and listed as fold‐increase on either TCP versus ECM Plus or vice versa (*p* < 0.05).

## Data Availability

The data that support the findings of this study are available from the corresponding author upon reasonable request.
