# Supplementary material for: Aging Adipose‐Derived Mesenchymal Stem Cells, Cultured on a Native Young Extracellular Matrix, Are Protected From Senescence and Apoptosis Along With Increased Expression of HLA‐DR and CD74 Associated With PI3K Signaling
Source: Aging Cell. 2025 Aug 5;24(9):e70165. doi: 10.1111/acel.70165 (PMC12419859; doi:10.1111/acel.70165)
Supplement: Supplementary file 1 — Appendix S1. [file ACEL-24-e70165-s003.pdf]

AGING CELL AUTHOR CHECKLIST. ***Authors should submit this checklist together with their manuscript. Please ensure that you have read the Author Guidelines in detail before submission.***

**Title**	Aging Adipose-derived Mesenchymal Stem Cells, Cultured on a Native Young Extracellular Matrix, Are Protected from Senescence and Apoptosis Along with Increased Expression of HLA-DR and CD74 Associated with PI3K Signaling
**Authors**	A.O. Gonzalez, P.A. Abdul Azees, J.P. Chen, M. Marinkovic, B. Cao, T. Liang, P. Hu, C.-K. Yeh, D.D. Dean, Y. Bai, X.-D. Chen
**Manuscript Type**	Research article
**Total Character Count (including spaces)[1]**	49,818
**Word count of Summary[2]**	247
**Number of papers cited in the References[3]**	44
**Listing of all Tables (Table1, Table 2 etc)[4]**	Table S1

**Figure specifications (please complete one row per figure)[5]** *Figure no.*	Colour *([redacted]/no)*	Greyscale *(yes/no)*	Black and white *(yes/no)*	Single column (80mm) *(yes/no)*	Double column (180mm) *(yes/no)*	Size of figure at full scale (mm x mm) *(insert details)*	Smallest font size used in the figure at full scale (minimum 6pt) *(insert details)*
Figure 1	Y				Y	180 X 250	8 pt
Figure 2	Y				Y	180 X 250	8 pt
Figure 3	Y				Y	180 X 250	8 pt
Figure 4	Y				Y	180 X 250	8 pt
Figure 5	Y				Y	180 X 250	8 pt
Figure 6	Y				Y	180 X 250	8 pt

[1] The maximum character count allowed is 50,000 (incl. spaces) for Primary Research Papers and Reviews, 10,000 for Short Takes.

[2] Summary should not exceed 250 words.

[3] Primary Research Papers can contain a maximum of two tables. If more are needed they should replace some of the Figures or can be placed in the Supporting Information.

[4] A maximum of 45 references is allowed for Primary Research Papers and 20 references for Short Takes.

[5] A Primary Research Paper may contain up to 6 figures and a Short Take up to 2 figures. Authors are encouraged to provide figures in the size they are to appear in the journal and at the specifications given.
