# Supplementary figures and images for: Aging Adipose‐Derived Mesenchymal Stem Cells, Cultured on a Native Young Extracellular Matrix, Are Protected From Senescence and Apoptosis Along With Increased Expression of HLA‐DR and CD74 Associated With PI3K Signaling
Source: Aging Cell. 2025 Aug 5;24(9):e70165. doi: 10.1111/acel.70165 (PMC12419859; doi:10.1111/acel.70165)

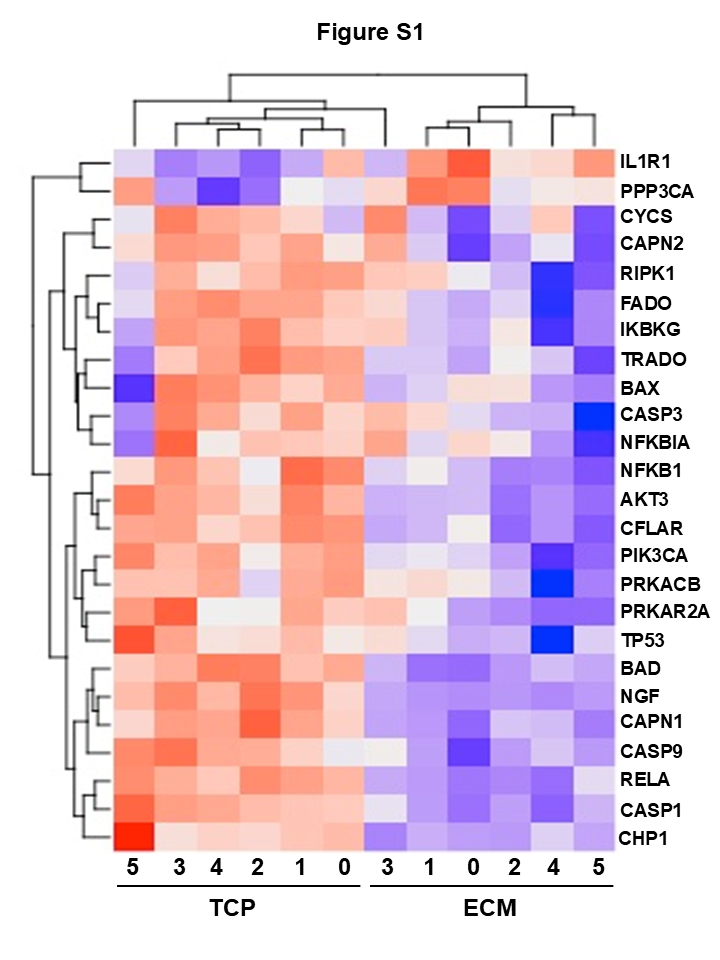

Supplement: Supplementary file 2 — Figure S1. An enlarged version of Figure 4D which displays up‐ or down‐ regulated apoptosis‐associated genes expressed by cells maintained on TCP versus ECM. [file ACEL-24-e70165-s002.tif]

## Figure S2

### Whole blots for figure 6A

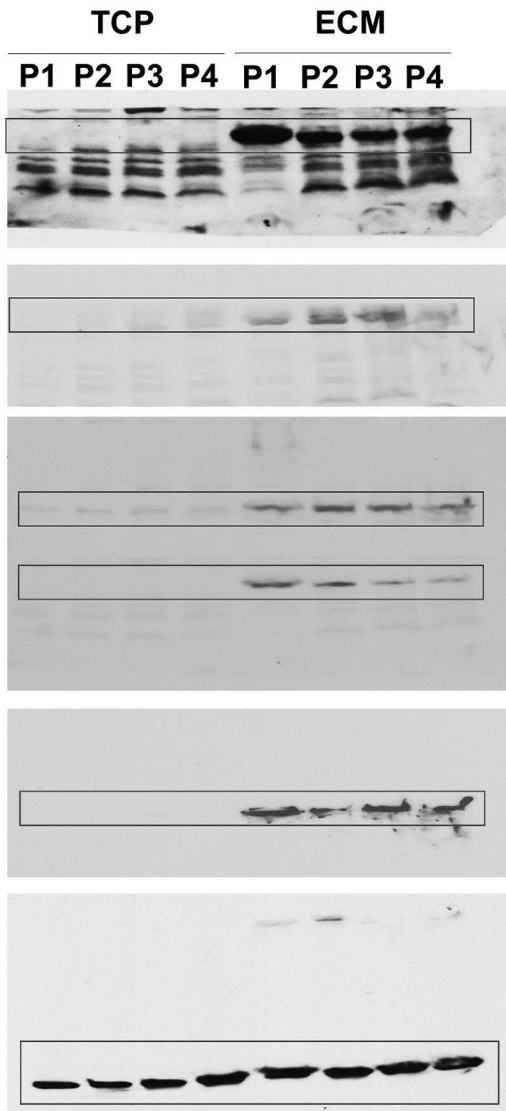

### Whole blots for figure 6B

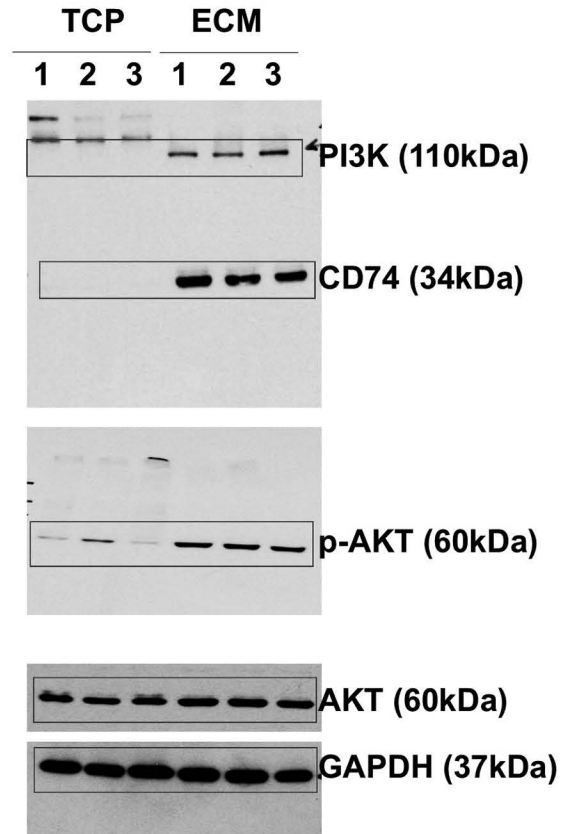

# Figure 2S

Whole blots for figure 6C

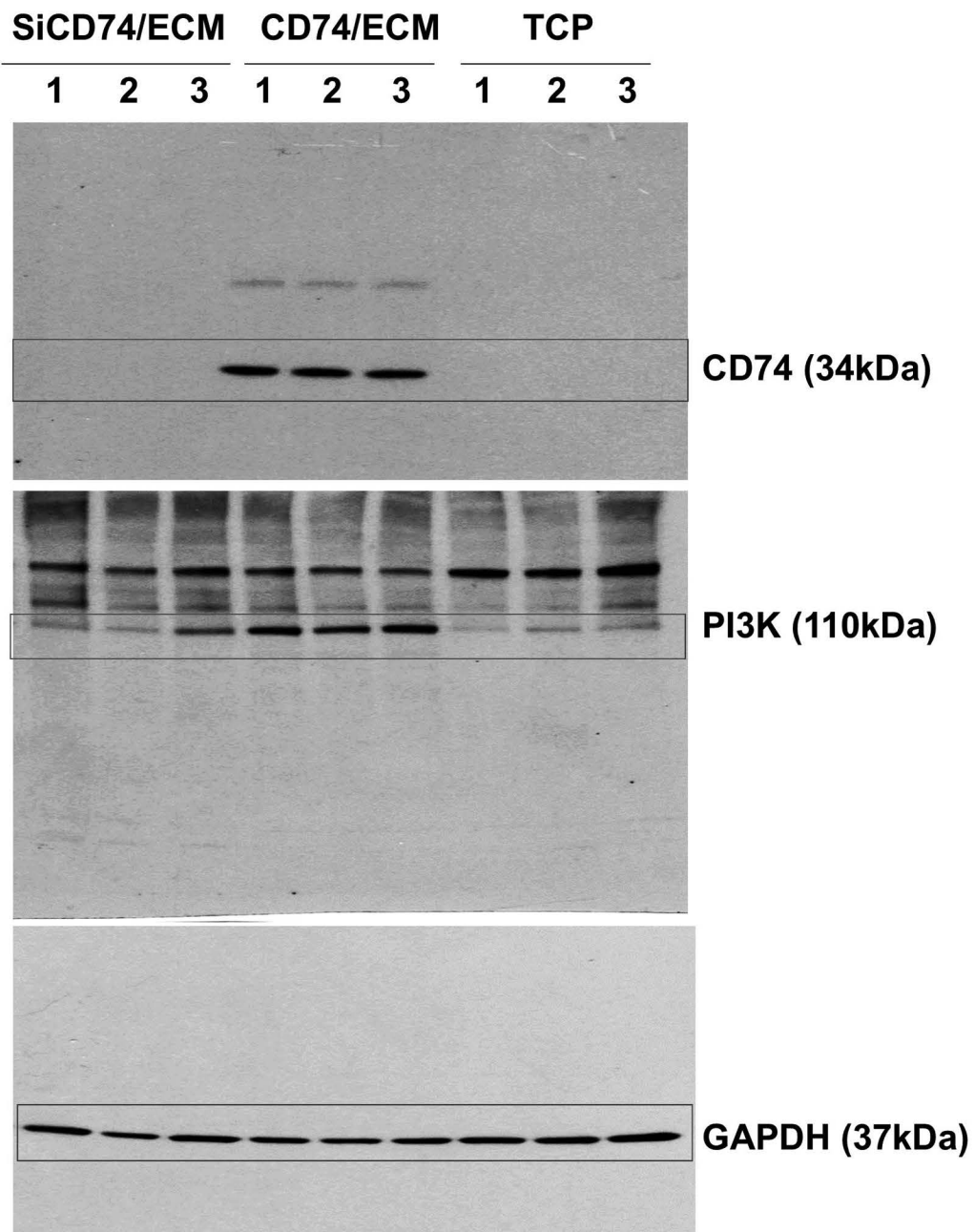

Supplement: Supplementary file 3 — Figure S2. Entire Wb blots used to create the blots shown in Figure 6A–C. The original blots showed some non‐specific bands and background. The indicated bands were identified by their molecular weight. [file ACEL-24-e70165-s001.pdf]

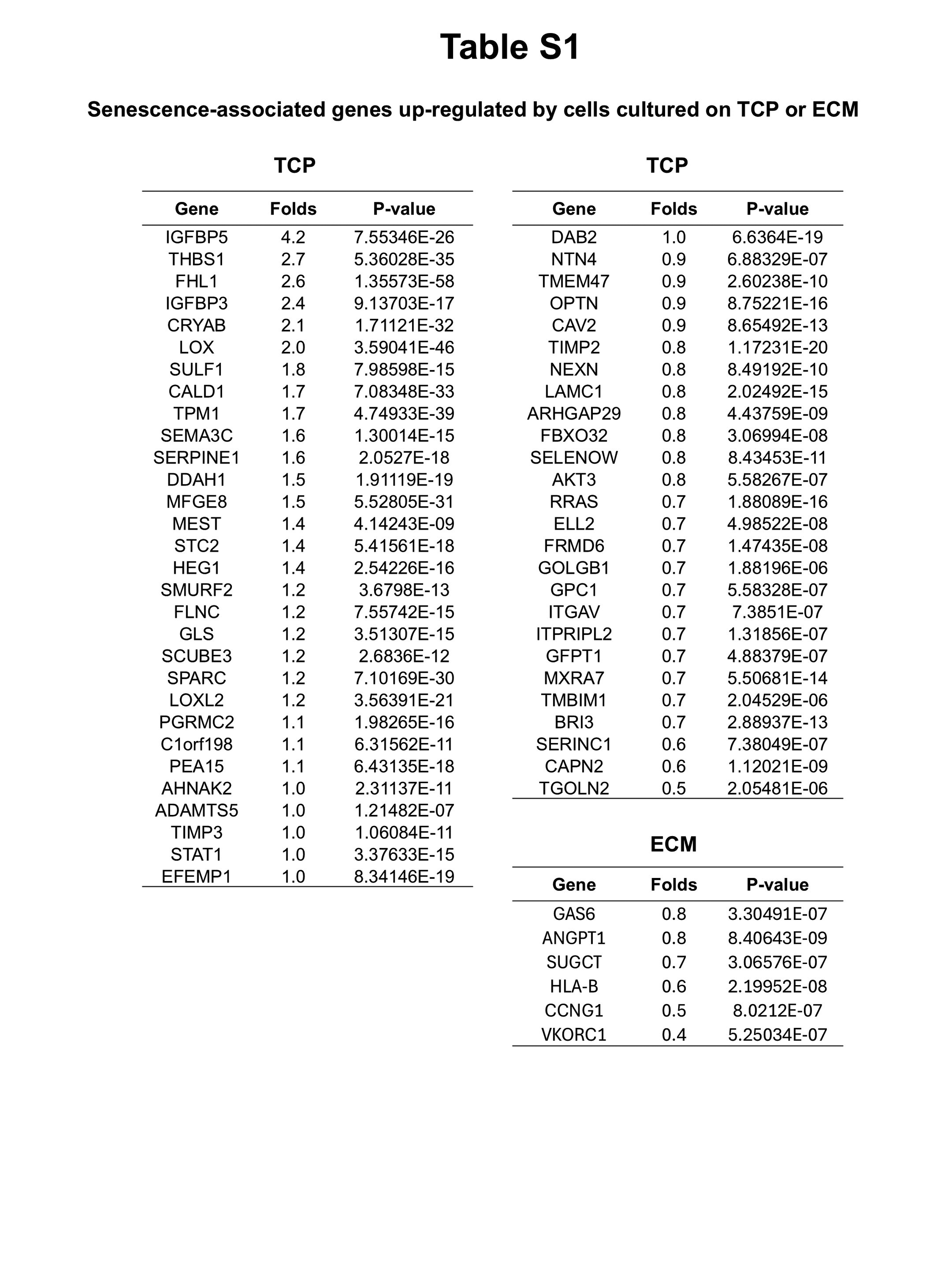

Supplement: Supplementary file 4 — Table S1. Senescence‐associated genes up‐regulated by cell culture on TCP or ECM. The senescence‐associated genes were identified in cluster 5 (Figures 4B,C) and listed as fold‐increase on either TCP versus ECM Plus or vice versa (p < 0.05). [file ACEL-24-e70165-s004.tif]
